# 肺鳞癌肿瘤浸润前沿细胞的EMT表型特点及临床意义

**DOI:** 10.3779/j.issn.1009-3419.2014.04.05

**Published:** 2014-04-20

**Authors:** 英华 宋, 才擎 张, 智新 曹, 嘉雯 徐, 令成 王, 晓燕 林

**Affiliations:** 1 250014 济南，山东大学附属山东省千佛山医院呼吸内科 Department of Respiratory Medicine, Shandong Provincial Qianfoshan Hospital, Shandong University, Ji'nan 250014, China; 2 250021 济南，山东大学附属省立医院病理科 Department of Pathology, Shandong Provincial Hospital Affiliated to Shandong University, Ji'nan 250021, China; 3 250031 济南，济南市第四人民医院呼吸内科 Department of Respiratory Medicine, the Forth People's Hospital of Jinan, Ji'nan 250031, China

**Keywords:** 肺鳞状细胞癌, 肿瘤浸润前沿, E-cadherin, β-catenin, Vimentin, Lung squamous cell carcinoma, Invasive tumor front, E-cadherin, β-catenin, Vimentin

## Abstract

**背景与目的:**

肿瘤浸润前沿（invasive tumor front, ITF）细胞是指肿瘤与宿主组织交界处的细胞或浸润的细胞团，对判断患者预后具有较高的价值。本研究旨在探讨肺鳞状细胞癌（squamous cell carcinoma, SCC）ITF细胞的上皮-间叶转化（epithelial-mesenchaymal transition, EMT）表型特点，并分析与临床病理特征和预后的关系。

**方法:**

采用免疫组织化学SP法检测104例肺SCC ITF细胞中上皮性标志物E-cadherin/β-catenin和间叶性标志物vimentin的表达。

**结果:**

E-cadherin在53.8%（56/104）的肺SCC ITF细胞中表达下调，较非ITF细胞表达降低（*P*=0.04），而vimentin在42.3%（44 /104）ITF细胞中表达，较非ITF肿瘤细胞表达升高；两者均与肿瘤浸润方式、肺门淋巴结转移和患者预后有相关性（*P* < 0.01）。β-catenin在肺SCC ITF细胞的表达阳性率为67.3%（70/104），低于非ITF细胞（*P* < 0.01），在ITF细胞呈胞质和胞核阳性表达，并与肺门淋巴结转移密切相关。

**结论:**

肺SCC ITF细胞中E-cadherin/β-catenin表达缺失和vimentin高表达可能与患者的不良预后有关。

## Abstract (en)

**Background and objective:**

The invasive tumor front (ITF) refers to cells or invasive nests in the junctional region of a tumor and its host. The ITF contains the most invasive cells of a tumor, and has a high prognostic value in carcinoma. The aim of this study is to investigate the epithelial-mesenchymal transformation phenotype in ITF cells of lung squamous cell carcinoma (SCC), and analyze the relationship between clinicopathological features and clinical outcomes of patients.

**Methods:**

Semiquantitative immunohistochemistry was used to examine the expression of epithelial markers (E-cadherin and β-catenin) and mesenchymal marker (vimentin) in 104 lung SCC tumor tissues.

**Results:**

A decrease in E-cadherin expression in ITF cells was observed in 56 of 104 (53.8%) tumors from patients. This result was markedly lower than that of non-ITF cells, which eventually developed metastatic tumors and were also associated with death (*P*=0.04). Vimentin expression was observed in 44 of 104 (42.3%) ITF cells, which was much higher than that of non-ITF cells. The downregulation of E-cadherin and overexpression of vimentin were associated with tumor invasive pattern, lymphatic metastasis, and poor prognosis (*P* < 0.01). The expression of β-catenin was 67.3% (70/104) in ITF cells. Moreover, ITF cells showed more nuclear and plasma-positive cells, which were closely associated with metastasis (*P* < 0.01).

**Conclusion:**

The loss in expression of E-cadherin/β-catenin and overexpression of vimentin in ITF cells may be associated with poor prognosis of lung SCC patients.

肺鳞状细胞癌（squamous cell cancer, SCC）是非小细胞肺癌（non-small cell lung cancer, NSCLC）的主要病理组织学类型之一，其发病率在全球范围内呈逐年升高趋势。虽然早期肺SCC可以治愈，但进展期患者的预后仍较差，局部复发和远处转移是其主要原因。因此，寻找可靠的预测复发及转移的分子标记物对于改善肺SCC的预后意义重大^[1]^。

肿瘤浸润前沿（invasive tumor front, ITF）定义为位于肿瘤与宿主组织和器官交界处的3层-6层细胞或散在浸润的细胞团。研究^[2]^证实，ITF细胞与肿瘤中心部分细胞的生物学特点迥异，对判断肿瘤患者预后具有较高的参考价值。近年来研究发现，ITF细胞是发生上皮-间质细胞转化（epithelial-mesenchaymal transition, EMT）最为明显的细胞，其特点是肿瘤细胞失去上皮细胞表型（E-cadherin），或者是获得了间叶细胞表型（vimentin），从而浸润性增强并促进肿瘤恶性进展。其中，E-cadherin/β-catenin复合体在调控肿瘤的EMT过程中参与了多种人类实体瘤的浸润、转移过程，但在ITF细胞中的表达意义报道不多^[3-5]^。本研究旨在探讨E-cadherin/β-catinin复合体和vimentin在肺SCC ITF细胞中的表达，分析ITF细胞的EMT表型与临床病理特点的关系以及在预测SCC患者预后中的价值。

## 1 对象与方法

### 1.1 组织学标本

收集2011年1月-2012年12月间山东省立医院胸外科手术切除的肺SCC标本104例，采集患者完整的临床病理学资料。由两名病理学医生依据WHO（2004年）标准对HE切片进行复审组织学诊断。所有患者均进行了肿瘤切除以及肺门淋巴结清扫术，并在术前未进行放化疗。相关临床病理资料见表 1。

**1:** E-cadherin、*β*-catenin和vimentin在肺SCC肿瘤中央细胞及ITF细胞的表达 Comparetion between E-cadherin, *β*-catenin and vimentin expression of ITF cells and tumor central cells

Group	E-cadherin					*β*-catenin					Vimentin			
	N	L	H	*P*		N	L	H	*P*		N	L	H	*P*
Non-ITF	14	20	70	0.008		7	9	88	0.013		80	16	8	0.008
ITF	26	30	48			13	21	70			60	24	20	
N: negative; L: low expression; H: high expression; ITF: invasive tumor front. SCC: squamous cell carcinoma.														

### 1.2 组织病理学评估

根据分化程度将肺SCC分为高分化、中分化和低分化3组。根据TNM分期分为Ⅰ期、Ⅱ期、Ⅲ期和Ⅳ期。肿瘤的浸润方式通过正常组织-肿瘤组织交界处评价，根据Bryne’s分类，浸润方式分为4型：1型为推挤型；2型为肿瘤为指状浸润或分离的大肿瘤细胞岛；3型为浸润的肿瘤岛由大于15个细胞组成；4型为浸润肿瘤细胞岛小于15个细胞，包括条索样和单个细胞浸润^[6]^。

### 1.3 免疫组织化学染色

采用SP法，组织切片经脱蜡及梯度酒精水化，3%过氧化氢H_2_O_2_封闭内源性的过氧化物酶，0.01 M枸橼酸钠缓冲液（pH6.0）微波加热进行抗原修复。加入一抗室温孵育60 min，山羊抗鼠二抗室温孵育30 min。抗体为E-cadherin单克隆抗体、β-catenin单克隆抗体和vimentin多克隆抗体（北京中杉生物技术公司）。切片PBS冲洗3次，DAB显色，苏木素复染。免疫反应结果经2名病理学医师在未知临床数据的情况下分别进行判断。癌旁肺组织做为阳性对照，PBS代替一抗作为阴性对照。

### 1.4 染色结果判断

选择典型的阳性染色区域，高倍镜下观察5个视野，每个视野计数100个细胞，总共计数500个细胞，然后计算出阳性百分率。根据染色强度和分布进行半定量评分：免疫组化分数=染色强度得分×阳性率得分。染色强度分为：阴性为0分，弱阳性1分，中等阳性2分和强阳性3分。阳性细胞比率分为：阴性为0分， < 10%为1分，11%-50%为2分，51%-80%为3分，≥81%为4分。根据最终得分将染色结果分为：阴性，为0分；1分-4分为低表达，≥4分为高表达。肿瘤浸润前沿细胞的免疫组化染色结果判断也按照此标准进行^[7]^。

### 1.5 随访和统计学分析

104例患者的随访时间为52周-144周，中位随访时间为106周。总生存率和无病生存率应用*Kaplan-Meier*曲线计算，用*Log-rank*检验对照。采用单因素和多因素*Cox*比例回归模型分析预后因素。临床病理学参数和E-cadherin、β-catenin和vimentin的表达关系应用χ^2^检验。数据分析应用SPSS 16.0 for windows统计分析软件。*P* < 0.05为差异有统计学意义。

## 2 结果

### 2.1 EMT蛋白在肺SCC

ITF细胞中的表达E-cadherin在正常肺泡上皮呈胞膜强阳性表达，在SCC中心部分肿瘤细胞表达降低或缺失（33.0%, 34/104），在ITF细胞中表达缺失率（53.8%, 56/104），较前两者明显降低（*P* < 0.05）。β-catenin在正常肺泡上皮呈胞膜阳性，中心肿瘤组织阳性率为84.6%（88/104），而ITF细胞的阳性率为67.3%（70/104），较正常组织和非ITF细胞均明显降低（*P*=0.01），部分细胞还出现了胞质和细胞核表达（20.0%，21/104）。Vimentin在正常肺组织上皮细胞不表达，但在23.1%（24/104）的SCC非ITF细胞的肿瘤组织强弱不等的阳性表达，而在ITF的细胞阳性率明显增加（42.3%，44/104高表达）（*P*=0.008）（图 1，表 1）。

**1:**
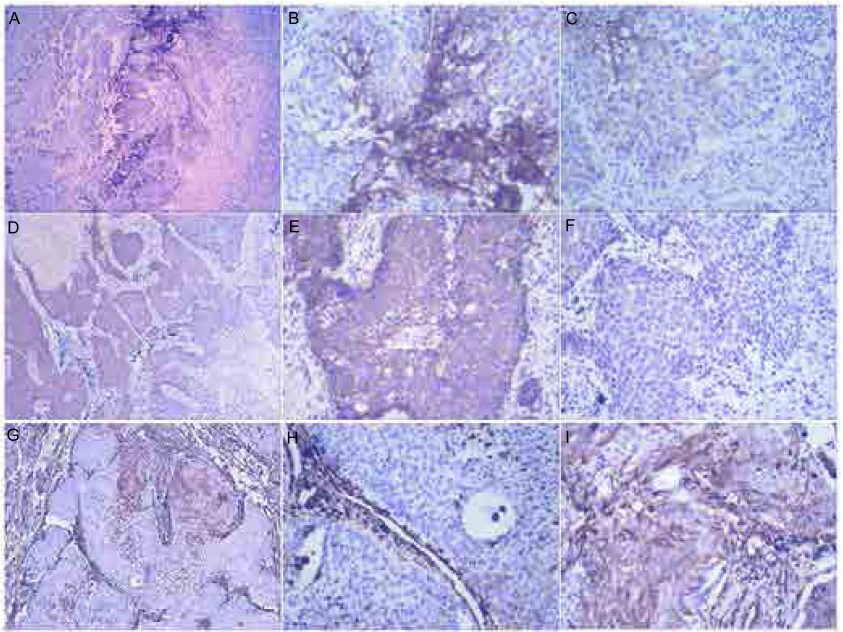
EMT相关蛋白在肺SCC中的表达。E-cadherin在肺SCC组织中的表达情况（A），在中央肿瘤组织胞膜强阳性（B），而ITF细胞中为阴性（C）；*β*-catenin在肺SCC组织中的表达情况（D），显示在肿瘤中央细胞胞膜和胞浆阳性（E），而ITF组织中为阴性（F）；vimentin在肺SCC组织中的表达（G），显示肿瘤中央细胞为阴性表达（H），而在ITF组织中高表达（I）（HE染色，A、D和G ×100倍；B、C、E、F、H、I×400倍） EMT protein expression was examined by immunohistochemistry in lung SCC. Expression of E-cadherin in lung SCC (A): E-cadherin was strong positive membranous staining (B), but negative was found in ITF cells (C); Expression of *β*-catenin in lung SCC (D), *β*-catenin was membranous or cytoplasmic positive staining (E), but negative in ITF cells (F); Expression of vimentin in lung SCC (G): vimentin was cytoplasmic positive staining (H), but negative in ITF cells (I). (A, D, H, ×100; B, C, E, F, H, I, ×400). EMT: epithelial-mesenchymal transition

### 2.2 SCC ITF细胞中E-cadherin、β-catenin和vimentin表达与各临床病理特征的关系

E-cadherin、β-catenin和vimentin在SCC ITF细胞的表达与患者的性别、年龄、肿瘤部位、肿瘤大小、组织学分化程度和临床分期均无相关性。ITF细胞中E-cadherin低表达和vimentin高表达在3型、4型浸润方式的ITF细胞中更为明显（*P*分别为0.01和0.02）。β-catenin在ITF细胞的表达与浸润方式无关，但是在3型、4型浸润方式中出现细胞核/质异常染色的比率（27.9%, 17/61）明显高于1型、2型浸润方式（15%, 6/40）（*P*=0.04）。此外，三者在ITF细胞中的表达均与肺门淋巴结转移和肿瘤复发相关。ITF细胞中E-cadherin下调组，β-catenin下调组和Vimentin上调组出现淋巴结转移率与对照组相比（66.1% *vs* 45.8%; 73.5% *vs* 48.6%; 70.0% *vs* 48.4%），具有明显差异（*P*分别为0.04、0.02和0.03）。ITF细胞中E-cadherin低表达组，β-catenin低表达组和vimentin高表达组出现肿瘤复发率与对照组相比（35.7% *vs* 18.8%; 41.2% *vs* 21.4%; 42.5% *vs* 18.8%），差异明显（*P*分别为0.04、0.04和0.01）。分析三者间的表达关系显示，E-cadherin的表达与β-catenin正相关（*P*=0.01），与vimentin表达呈负相关（*P*=0.035）；而vimentin和β-catenin两者的表达不相关（表 2）。

**2:** E-cadherin、*β*-catenin和vimentin三者在SCC ITF细胞的表达与各种临床病理特征的关系 Relationship between E-cadherin, *β*-catenin and vimentin expression levels of ITF cells and clinical variables of SCC

Group	*n*	E-cadherin				*β*-catenin				vimentin		
		L	H	*P*		L	H	*P*		L	H	*P*
Gender				0.60				0.11				0.76
Male	72	34	38			20	52			45	27	
Female	32	22	10			14	18			19	13	
Age (yr)				0.82				0.49				0.87
≤50	38	21	17			14	24			23	15	
> 50	66	35	31			20	46			41	25	
Tumor location				0.85				0.79				0.94
Central	81	44	37			27	54			50	31	
Peripheral	23	12	11			7	16			14	9	
Tumor size (cm)				0.14				0.07				0.45
≤3	62	37	25			16	46			40	22	
> 3	42	19	23			18	24			24	18	
Histological differentiation				0.07				0.40				0.53
Well	27	15	12			9	18			19	8	
Moderate	54	25	29			15	39			31	23	
Poor	23	16	7			10	13			14	9	
Invasion pattern				0.01^*^				0.52				0.02^*^
1	18	6	12			6	12			13	5	
2	22	9	13			10	12			17	5	
3	35	18	17			10	25			23	12	
4	29	23	6			8	21			11	18	
TNM stage				0.44				0.30				0.12
Ⅰ	41	24	17			11	30			29	12	
Ⅱ/Ⅲ	63	32	31			23	40			35	28	
Lymph node status				0.04^*^				0.02^*^				0.03^*^
N (+)	59	37	22			25	34			31	28	
N0	45	19	26			9	36			33	12	
Recurence				0.04^*^				0.04^*^				0.01^*^
Yes	29	20	9			14	15			12	17	
No	75	36	39			20	55			52	23	
*n*: number of patients; L: low expression, including negative and low expression; H: high expression; N0: no nodal metastasis; N(+): nodal metastasis. ^*^: *P* < 0.05.												

### 2.3 生存分析

根据最后一次随访的结果，24例（23.1%）患者无瘤存活，20例（19.2%）患者带瘤生存，60例患者（57.7%）死于肿瘤复发。*Kaplan-Meier*法分析显示ITF E-cadherin高表达组和vimentin低表达组的患者预后分别较ITF E-cadherin低表达组和vimentin高表达组好（*P* < 0.01）（图 2）。根据单因素的Cox比例风险回归模型分析，肿瘤大小（*P*=0.04）、淋巴结状态（*P*=0.04）、ITF细胞的vimentin（*P* < 0.01）和E-cadherin的表达水平（*P* < 0.01）与总生存率相关。在多因素*Cox*比例回归分析，ITF细胞的vimentin表达（*P*=0.042）和E-cadherin表达（*P*=0.016）与患者的总生存率和无病生存密切相关。

**2:**
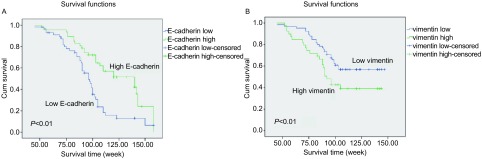
ITF细胞中EMT表型与患者预后的关系。A：E-cadherin；B：vimentin Survival curve by expression of E-cadherin and vimentin in lung SCC. A: E-cadherin; B: vimentin

## 3 讨论

所谓ITF是指位于肿瘤与宿主组织或器官交界处最前沿的3层-6层肿瘤细胞或分散的细胞团，该部分肿瘤细胞较之其他部分肿瘤细胞分化更差，其形态和功能特征更能反映肿瘤的生物多形性及侵袭性，众多研究发现多种肿瘤的预后与ITF细胞的生物学特性密切相关。

E-cadherin是上皮细胞中表达的一种钙依赖性跨膜糖蛋白。正常情况下E-cadherin的胞质内区段与β-catenin形成复合体，作为细胞-细胞连接促进细胞间的粘附性，在维持细胞极性和组织学结构中发挥重要作用。已证实多种恶性上皮性肿瘤中E-cadherin/β-catenin复合体表达下调与肿瘤浸润转移和不良预后有关^[3-5]^，但有关肺SCC ITF细胞的研究少见。本研究结果显示E-cadherin在肺SCC肿瘤组织尤其是在ITF细胞中表达较正常组织降低，并与肿瘤的浸润方式、肺门淋巴结转移和复发等因素密切相关，单因素及多因素分析均显示其是肺SCC的预后标记。这与Choi等^[8]^的研究结果一致。有关SCC ITF细胞EMT表型的研究目前多见于口腔SSC肿瘤中，Kim等^[9]^检测到83例口腔SCC ITF细胞中E-cadherin的表达降低并与淋巴结转移和患者预后有关。而Wang等^[10]^研究认为E-cadherin在肺SCC ITF细胞的表达缺失并不是无瘤生存期的预后指标。

β-catenin是一种双向功能的连接素，正常位于胞膜。在致癌因子的作用下β-catenin聚集于细胞质和/或胞核中，与TCF/LEF（T cell factor/lymphoid enhancer factor）结合启动*cyclin D1*和*c-myc*等基因转录，加快肿瘤侵袭转移^[11]^。β-catenin在肺癌ITF中的表达鲜见报道。本研究证实肺SCC的ITF细胞中β-catenin表达明显低于正常组织和非ITF肿瘤中心组织，同时观察到出现胞核异常表达，并且与肺门淋巴结转移和肿瘤复发相关。Choi等^[8]^研究也认为β-catenin的表达缺失与肺腺癌的预后相关，而与SCC的预后无关。Sasaya等^[12]^研究了62例口腔SCC中β-catenin的表达，显示ITF细胞β-catenin表达降低出现胞质/胞核着色，并与不良预后有关。Zhang等^[13-15]^认为肿瘤组织β-catenin的表达缺失与肺癌患者无病生存期有关，而我们的研究中发现两者并无相关性，分析原因可能与样本数量和判断标准不同有关。目前，有关β-catenin表达及预后意义的研究结论还有待大样本深入研究^[16, 17]^。

Vimentin是一种间质细胞的标志物，其表达与多种恶性肿瘤如乳腺癌、肝癌、结肠癌和前列腺癌的恶性表型和不良预后相关^[18]^。Soltermann等^[19]^发现vimentin的启动子是β-catenin/TCF细胞通路的靶点，共同参与了细胞侵袭和迁移。本文研究结果表明vimentin在肺SCC的ITF细胞表达较非ITF细胞明显增加，vimentin强阳性表达在肿瘤的ITF细胞中是常见的现象，并与肿瘤的浸润方式、肺门淋巴结转移和肿瘤复发密切相关。我们应用单因素或多因素分析显示，vimentin的过表达与短的总生存时间和无病生存时间有关，提示肿瘤ITF细胞vimentin高表达是提示不良预后的分子标志物之一。

通过分析E-cadherin/cantenin和vimentin三者的表达关系，发现在肿瘤的ITF细胞E-cadherin和β-catenin的表达呈正相关关系；vimentin和E-cadherin表达呈负相关，vimentin表达与E-cadherin丢失是细胞间叶化的特征，这些发现进一步支持了vimentin可下调E-cadherin表达的结论^[20]^。然而vimentin表达与β-catenin无关，提示可能是β-catenin/TCF通路可能在调控vimentin表达的过程中未发挥作用。

总之，E-cadherin/β-catenin与vimentin在肺SCC ITF细胞中的表达与患者的预后密切相关，这些指标的异常表达可预测患者的不良预后，其具体的预后价值尚需大样本资料的进一步研究。
